# Pulmonary embolism detection without intravenous contrast using electron density and Z-effective maps from dual-energy CT

**DOI:** 10.1093/radadv/umae025

**Published:** 2024-10-24

**Authors:** Tommaso D’Angelo, Simone Barbera, Velio Ascenti, Giuseppe Cicero, Simone Terrani, Damiano Caruso, Andrea Laghi, Federico Fontana, Massimo Venturini, Filippo Piacentino, Christian Booz, Thomas J Vogl, Ibrahim Yel, Maria Adele Marino, Silvio Mazziotti, Giorgio Ascenti

**Affiliations:** Diagnostic and Interventional Radiology Unit, BIOMORF Department, University Hospital “Policlinico G. Martino”, 98100 Messina, Italy; Department of Radiology and Nuclear Medicine, Erasmus MC, 3015 GD Rotterdam, The Netherlands; Diagnostic and Interventional Radiology Unit, BIOMORF Department, University Hospital “Policlinico G. Martino”, 98100 Messina, Italy; Department of Radiology, Policlinico Universitario, University of Milan, 20133 Milano, Italy; Diagnostic and Interventional Radiology Unit, BIOMORF Department, University Hospital “Policlinico G. Martino”, 98100 Messina, Italy; Clinical Application Department, Philips Healthcare, 20126 Milano, Italy; Department of Medical Surgical Sciences and Translational Medicine, Sapienza University of Rome, 00185 Rome, Italy; Department of Medical Surgical Sciences and Translational Medicine, Sapienza University of Rome, 00185 Rome, Italy; Diagnostic and Interventional Radiology Unit, Circolo Hospital, ASST dei Sette Laghi, 21100 Varese, Italy; Diagnostic and Interventional Radiology Unit, Circolo Hospital, ASST dei Sette Laghi, 21100 Varese, Italy; Diagnostic and Interventional Radiology Unit, Circolo Hospital, ASST dei Sette Laghi, 21100 Varese, Italy; Department of Diagnostic and Interventional Radiology, University Hospital Frankfurt, 60590 Frankfurt am Main, Germany; Department of Diagnostic and Interventional Radiology, University Hospital Frankfurt, 60590 Frankfurt am Main, Germany; Department of Diagnostic and Interventional Radiology, University Hospital Frankfurt, 60590 Frankfurt am Main, Germany; Diagnostic and Interventional Radiology Unit, BIOMORF Department, University Hospital “Policlinico G. Martino”, 98100 Messina, Italy; Diagnostic and Interventional Radiology Unit, BIOMORF Department, University Hospital “Policlinico G. Martino”, 98100 Messina, Italy; Diagnostic and Interventional Radiology Unit, BIOMORF Department, University Hospital “Policlinico G. Martino”, 98100 Messina, Italy

**Keywords:** contrast-enhanced CT, contrast media, dual-energy CT, electron density, pulmonary embolism

## Abstract

**Purpose:**

This study aims to evaluate the feasibility of using electron density (ED) maps combined with Z-effective (Zeff) images obtained from unenhanced dual-layer dual-energy CT (dl-DECT) scans of the chest for the detection of pulmonary embolism (PE).

**Materials and methods:**

A retrospective analysis was conducted on consecutive patients who underwent for contrast-enhanced chest CT (CECT) clinically suspected of PE or acute aortic syndrome. These scans were performed on a single dl-DECT scanner between October 2021 and November 2023. To distinguish emboli from circulating blood, color-coded maps were generated from the ED dataset superimposed on Zeff images, which were acquired from the unenhanced phase. Two radiologists with different levels of expertise independently assessed the presence of PE in the generated ED-Zeff maps, blinded to CECT results, which served as the reference standard. Diagnostic accuracy of ED-Zeff maps was assessed for each reader.

**Results:**

The final study cohort comprised 150 patients, with 92 males (mean age: 68 ± 10 years, range: 47-93 years) and 58 females (mean age: 66 ± 15 years, range 38-89 years). ED-Zeff maps demonstrated high diagnostic performance, yielding accuracy, sensitivity, and specificity, respectively, of 86.67% (113/150, 95% CI, 80.16%-91.66%), 85% (17/20, 95% CI, 79.89%-92.19%), and 86.92% (113/130, 95% CI, 79.89%-92.19%). Ed-Zeff maps were able to identify PE in 85% of positive cases. Cohen’s kappa coefficient indicated excellent intra- and interobserver agreement (κ ≥ 0.9).

**Conclusion:**

ED maps combined with Zeff images from unenhanced dl-DECT scans represent a feasible tool for detecting PE and may prove useful in evaluating patients with contraindications to iodinated contrast.

**Abbreviations**

CECT = contrast-enhanced CT; CTA = CT angiography; CTPA = CT pulmonary angiography; DECT = dual-energy CT; dl-DECT = dual-layer dual-energy CT; ED = electron density; PE = pulmonary embolism; SBI = spectral base images; VMI = virtual monoenergetic image; Zeff = Z-effective

**Summary**

Electron density maps combined with Z-effective (atomic number) images obtained from unenhanced dual-energy CT scans represent a feasible diagnostic tool for detecting pulmonary embolism.

**Key Results**

- Electron density map is a dual-energy algorithm that describes the probability of an electron being present at a specific location around the atomic nucleus.
- The electron density map superimposed on Z-effective map, both derived from dual-energy CT, can distinguish circulating blood from embolus.
- These maps enable detection of pulmonary embolism without intravenous contrast.
- Non-contrast dual-energy CT may be useful in evaluating patients with contraindications to iodinated contrast for suspected pulmonary embolus.

## Introduction 

Contrast-enhanced CT (CECT) is currently considered the gold standard for diagnosing pulmonary embolism (PE).^1^ Technical advancements such as the advent of multidetector CT first permitted the acquisition of high-quality images demonstrating small sub-segmental pulmonary artery emboli. Subsequently, dual-energy CT (DECT), was shown to provide both morphological and functional pulmonary information in a single contrast-enhanced phase. This is achieved through the simultaneous demonstration of pulmonary emboli occluding the pulmonary vessels and the consequent perfusion defects in the affected lung parenchyma on iodine maps.^2^ DECT virtual monoenergetic imaging (VMI) also showed to boost the signal- and contrast-to-noise ratio of iodinated pulmonary vessels, in order that CT pulmonary angiography (CTPA) can be performed with a minimal amount of iodine.^3–5^

However, despite CT technical evolution, limitations may persist in patients with impaired renal function (ie, glomerular filtration rate below 30 mL/min per 1.73 m^2^) or allergies to contrast agent, which may still discourage clinicians from ordering CTPA. In the Prospective Investigation of Pulmonary Embolism Diagnosis II (PIOPED-2) study, out of 7284 enrolled patients, 272 were ineligible to CTPA due to contrast medium allergy and 1350 due to high creatinine values.^6^ Nonetheless, alternative imaging modalities such as MRI or scintigraphy may not be desirable options. Chest MRI is a not widely available and time-consuming technique that is not practically feasible in an emergency setting. Similarly, lung scintigraphy would not be able to provide an alternative diagnosis other than PE.

Dual-layer DECT (dl-DECT) using the raw image data obtained at high and low energy, allows for the generation of spectral base images (SBI), which can be processed through various spectral algorithms for application in diverse clinical scenarios.^7–9^ Among these, electron density (ED) and Z-effective (Zeff) maps have been the most recently introduced.^10^ ED maps express the likelihood of an electron being present at a specific location, and their applications have been used mainly in radiotherapy.^11–14^ Zeff color-coded maps reflect an estimation of the atomic number of a material.^10^ On the other hand, conventional single-energy CT can only provide a raw estimation of ED or Z-number of a material, by applying established correlations with Hounsfield units.^15^^,^^16^

Recently, a few cases in the literature have reported the capability of ED maps to demonstrate vascular and cardiac thrombi without the use of contrast medium.^17–19^ The purpose of this study is to investigate the diagnostic performance of ED maps combined with Zeff maps derived from dl-DECT for the detection of PE on contrast-free images.

## Materials and methods

### Study design and patient population

**Figure 1.**
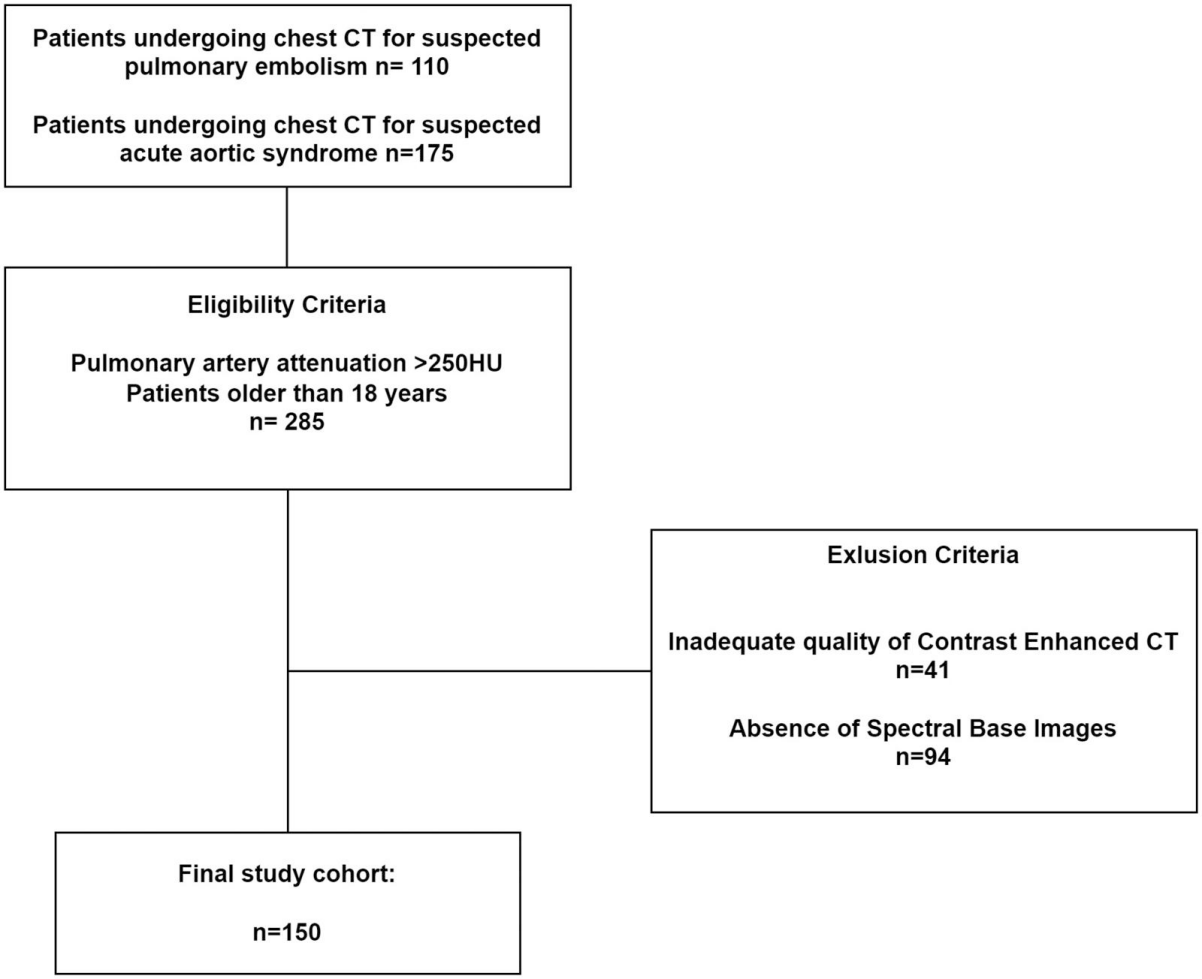
Flowchart of inclusion and exclusion criteria. The inclusion criterion >250 HU refers to 40 keV virtual monoenergetic images of the contrast-enhanced CT dataset.

A waiver for informed consent was obtained for this IRB-approved retrospective study. Between October 2021 and November 2023, a retrospective analysis was conducted on 285 consecutive patients with clinical suspicion of PE (110), or acute aortic syndrome (175), who underwent dl-DECT imaging of the chest, encompassing both unenhanced and contrast-enhanced phases (Figure 1).

**Figure 2.**
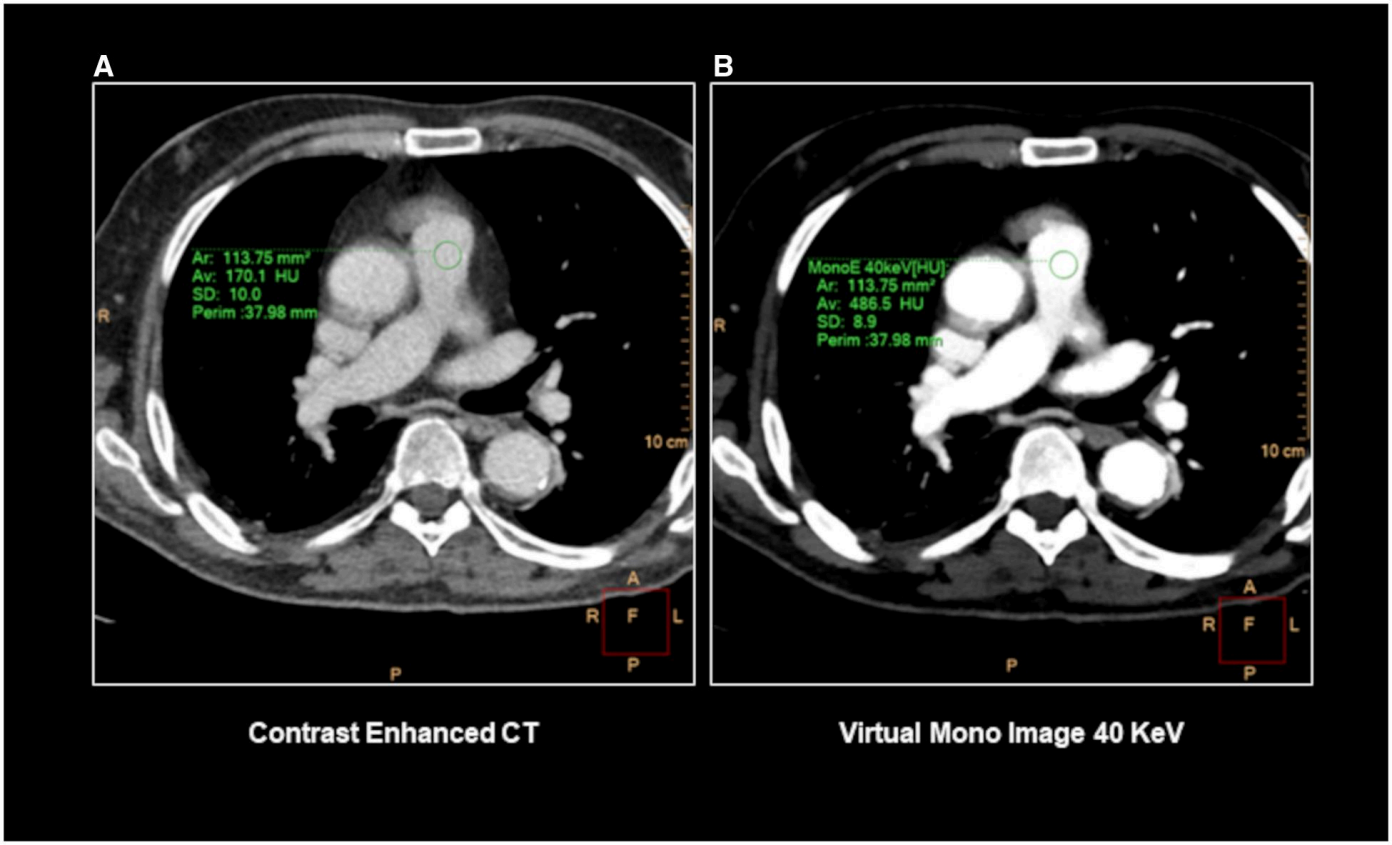
Example of conventional CECT (contrast-enhanced computed tomography) of the chest with poor contrast attenuation of pulmonary vessels (A). The use of VMI (virtual monoenergetic images) reconstruction at 40 keV (B) allows for increasing vascular attenuation (ie, 486 HU at the level of main pulmonary artery).

Given that not all examinations were conducted for clinical suspicion of PE, the primary eligibility criterion focused on the diagnostic quality of pulmonary vessels, evaluating quantitative and qualitative parameters. The main inclusion criterion was represented by pulmonary artery attenuation >250 HU, measured using a circular region-of-interest within the common pulmonary trunk, and adequate visualization of peripheral branches up to the sub-segmental pulmonary arteries.^20^^,^^21^ For this reason, VMI at 40 kilo electronvolts (keV) with optimized window settings were used in place of conventional CECT images (Figure 2) to achieve optimal conditions for detecting the presence of PE, exploiting increased iodine attenuation and improved image quality.^22–24^

Additional eligibility criteria included patients aged 18 years or older and the availability of raw data for spectral reconstructions in both unenhanced and post-contrast phases. Ultimately, 41 patients with inadequate-quality CECT images and 94 patients lacking SBI were excluded. The final study cohort consisted of 150 enrolled patients, 60 patients (40%) with clinical suspicion of PE and 90 patients (60%) with suspected acute aortic syndrome.

### dl-DECT image acquisition and reconstruction

All CT examinations were performed using dl-DECT (IQon Spectral CT, Philips Healthcare, Best, The Netherlands) with the following acquisition parameters: tube voltage 120 kVp; automatic modulation of mAs; nominal layer thickness 0.67 mm; scan interval: 0.34 mm; detector collimation: 64 × 0.625 mm; tube rotation time: 0.27 s; matrix 512 × 512; Field of View: variable according to patients’ characteristics. Typically, our study protocol includes an unenhanced phase and a post-contrast phase, employing a bolus-tracking technique with scan timing and region-of-interest adjusted to study either the pulmonary (CTPA) or systemic arterial circulation (CTA), based on the clinical suspicion. All patients received the standardized contrast administration protocol used in our institution, consisting of 1.3 mL/kg body weight of an intravenous non-ionic contrast medium (Iomeprol, 400 mg I/mL; Iomeron 400; Bracco, Milan, Italy) at a rate of 3-4 mL/s, followed by a 30 cc of saline solution at the same rate, using a power injector system.

Axial images were reconstructed using iterative reconstruction (iDose4), as suggested by the manufacturer, with a 2 mm slice thickness and 1 mm reconstruction interval. SBI data were automatically generated and sent to a dedicated workstation (IntelliSpace Portal v.8.0.2, Philips Healthcare, Best, Netherlands) to obtain superimposed ED and Zeff (ED-Zeff) color-coded maps from the unenhanced phase, while VMI reconstructions at 40 keV were generated from the contrast-enhanced phase.

### dl-DECT image analysis

Analysis was conducted on a per-patient basis using a binary approach (ie, the presence or absence of PE).

Preliminarily, a reader with more than 10 years of experience in cardiovascular imaging (T.D.) independently assessed the presence of PE to define a training cohort of 10 cases (5 positive and 5 negative), by only evaluating CECT datasets, blinded to ED-Zeff maps. Subsequently, 2 different readers—one with over 15 years of experience (G.A.) and the other with 3 years of experience (V.A.)—self-trained to identify PE on color-coded ED-Zeff maps by having access to their respective and coregistered CECT datasets. On CECT, the presence of PE was indicated by a visible contrast filling defect within the pulmonary arterial lumen. On ED-Zeff maps, the presence of PE was indicated by a visible difference in contrast within the pulmonary arterial lumen at the corresponding side and lobar/segmental level.

After a washout period of 2 weeks, both readers independently reviewed the study cohort by examining ED-Zeff maps while blinded to CECT datasets to identify the presence of PE, regardless of its anatomical site.

### Reference standard

A blinded consensus review of the study cohort was performed by the 2 readers (G.A. and V.A.) on the 40 KeV VMI CECT datasets, after a washout period of 3 weeks following image analysis. The review was conducted in randomized order to confirm or exclude the presence of PE. Additionally, the prevalence and distribution of pulmonary arterial lumen filling defects within the pulmonary arterial tree were also assessed (Table 1).

**Table 1.** Study cohort description and distribution of pulmonary emboli.

	All	CTA	CTPA
Patients, *N* (%)	150 (100)	90 (60)	60 (40)
Male, *N* (%)	92 (61)	58 (39)	34 (22)
Age mean, years (±SD)	68 ± 10		
BMI mean (±SD)	27 ± 5		
Female	58 (39)	32 (22)	26 (17)
Age mean, years (±SD)	66 ± 15		
BMI mean (±SD)	25.5 ± 7		
Pulmonary embolism, *N* (%)	20 (100)	10 (50)	10 (50)
Anatomical location			
Central, *N* (%)	6 (30)	3 (15)	3 (15)
Lobar, *N* (%)	5/20 (25)	1 (5)	4 (20)
Segmental, *N* (%)	9/20 (45)	6 (30)	3 (15)

Abbreviations: BMI = body mass index; CTA = CT angiography; CTPA = CT pulmonary angiography.

### Statistical analysis

Statistical analysis was performed using statistical software (Matlab, MathWorks v. R2023a, Natick, MA, USA). Data distribution was assessed with the Shapiro-Wilk test. Categorical variables were reported as numbers and percentages. Categorical variables were compared using Fisher’s exact test. A *P*-value <.05 was used to confirm a statistically significant difference. ED-Zeff maps diagnostic performance for each reader’s assessment of PE was calculated by a confusion matrix. Cohen’s kappa coefficient (κ) was used to calculate the intra- and interobserver agreement between the readers.

## Results

The final study cohort comprised 150 patients, with 92 males (mean age: 68 ± 10 years, range: 47-93 years) and 58 females (mean age: 66 ± 15 years, range 38-89 years) (Table 1). The attenuation of the common pulmonary trunk was higher than 350 HU for all cases (532.94 ± 100.62 HU).

According to the reference standard, the prevalence of PE was 13.33%, affecting 20 patients. The confusion matrix illustrates the results obtained by the most experienced reader (Figure S1), with the count of true positives (17, 11.33%), false positives (17, 11.33%), false negatives (3, 2.01%), and true negatives (113, 75.33%).

The sensitivity, specificity, and accuracy of ED-Zeff maps in detecting PE were 85% (95% CI, 62.11%-96.79%), 86.92% (95% CI, 79.89%-92.19%), and 86.67% (95 CI, 80.16%-91.66%), respectively. The positive predictive value (PPV) and negative predictive value (NPV) of the test were PPV = 50% (95% CI, 38.23%-61.77%) and NPV = 97.41% (95% CI, 92.98%-99.08%). Table 2 summarizes the diagnostic performance of ED-Zeff maps according to the experience of the readers. The Cohen’s kappa coefficient showed excellent intraobserver agreement for both readers (κ ≥ 0.90) and excellent interobserver reliability (κ = 0.91) for the identification of PE. The Fisher’s exact test indicated a significant association between ED-Zeff maps and the reference standard, for both readers (both *P* < 0.05).

**Table 2.** Overall and single readers’ diagnostic performance of ED-Zeff maps to detect PE according to years of experience in cardiovascular imaging.

	Sensitivity	Specificity	NPV	PPV
R1: >15 years	85	86.92	97.41	50
CVI experience	(82.11-96.79)	(79.89-92.19)	(92.98-99.08)	(38.23-61.77)
R2: <5 years	80	82.31	96.40	41.03
CVI experience	(56.34%-94.27)	(74.65-88.44)	(91.73-98.47)	(31.14-51.69)
Overall	82.50	84.85	96.97	45.21
	(67.22-92.66)	(79.94-88.95)	(94.22-98.43)	(37.48-53.17)

Data are shown as percentage (95% confidence interval).

Abbreviations: CVI = cardiovascular imaging; ED-Zeff = electron density-Z-effective; NPV = negative predictive value; PE = pulmonary embolism; PPV = positive predictive value.

## Discussion

Our study assesses the diagnostic performance of contrast-free DECT in detecting PE. The color-coded ED and Z-effective maps (ED-Zeff maps) offer a simple evaluation method, showing excellent intra- and interobserver agreement and a sensitivity of 85% and specificity of 86.92% compared to contrast-enhanced CT. These findings highlight the potential for DECT ED-Zeff maps in evaluating patients with contraindication to contrast for pulmonary emboli.

Emboli consist of different proportions of cellular debris, platelets, red blood cells, and fibrin. CT attenuation of emboli increases proportionally with the erythrocytes content, while it decreases when the fibrin component is predominant, as in venous emboli. Consequently, distinguishing between plasma and pure fibrin emboli, such as those occurring in PE, may be challenging on conventional CT images due to their similar attenuation numbers.^25^^,^^26^ However, the increase in attenuation results from the rise in material density, and consequently, its ED.

Phantom studies have indicated that discrepancies between plasma and emboli are better demonstrated using Compton scattering techniques.^27^ Moreover, several studies have reported the use of ED maps to potentially distinguish circulating blood from emboli without contrast injection.^14–19^ Mochizuki et al incidentally detected ventricular thrombi using spectral evaluation with ED maps, and acute iliac artery occlusion in a COVID-19 patient.^17^^,^^18^ Bae et al diagnosed PE in a 90-year-old man using spectral evaluation through ED maps, subsequently confirmed by CECT.^19^

**Figure 3.**
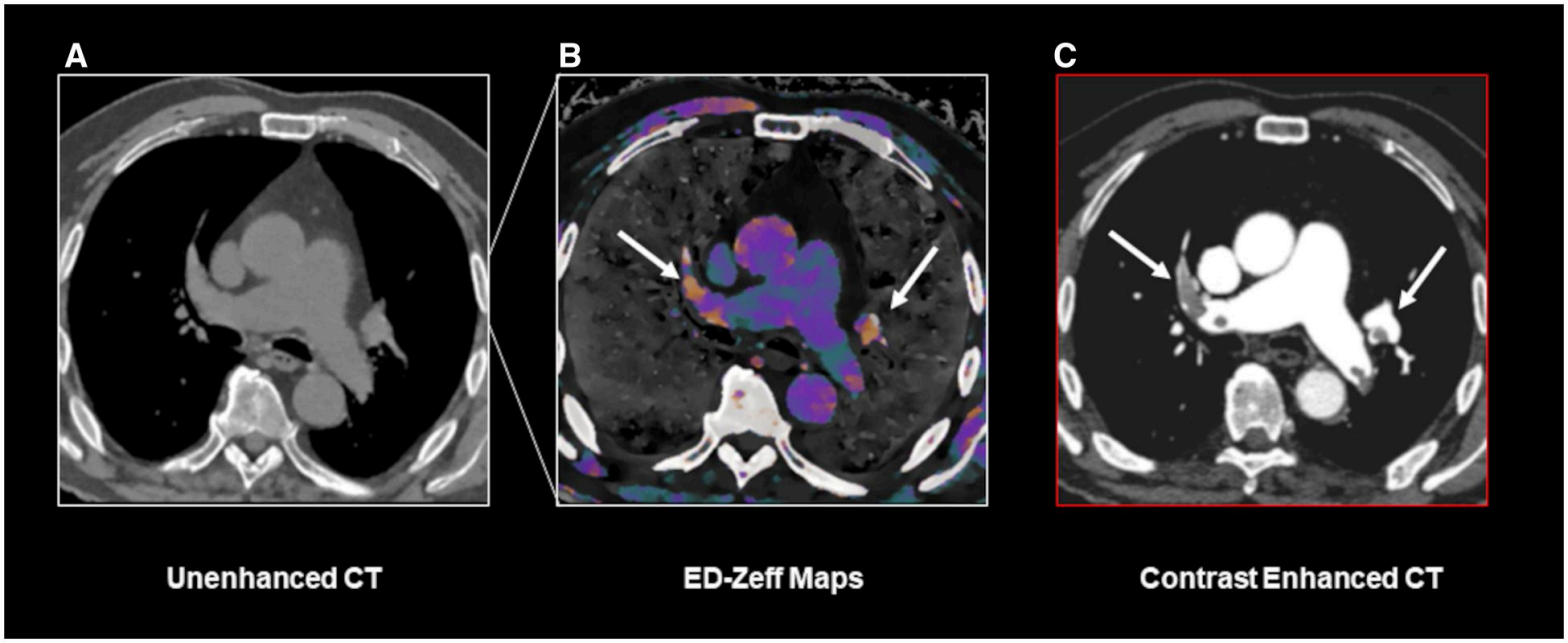
A true positive case of PE identified using the ED-Zeff (electron density-Z-effective) map. An 83-year-old male patient presenting to our department with dyspnea, tachypnea, and chest pain. Unenhanced CT scan of the chest (A) shows only mild dilatation of main pulmonary artery. ED-Zeff map (B), derived from the unenhanced CT scan, shows the presence of high signal spots (arrows) within the right and left pulmonary arteries (arrows). The subsequently performed CECT (contrast-enhanced computed tomography) (C) confirms the presence of pulmonary emboli (arrows) at the same location as shown in B.

**Figure 4.**
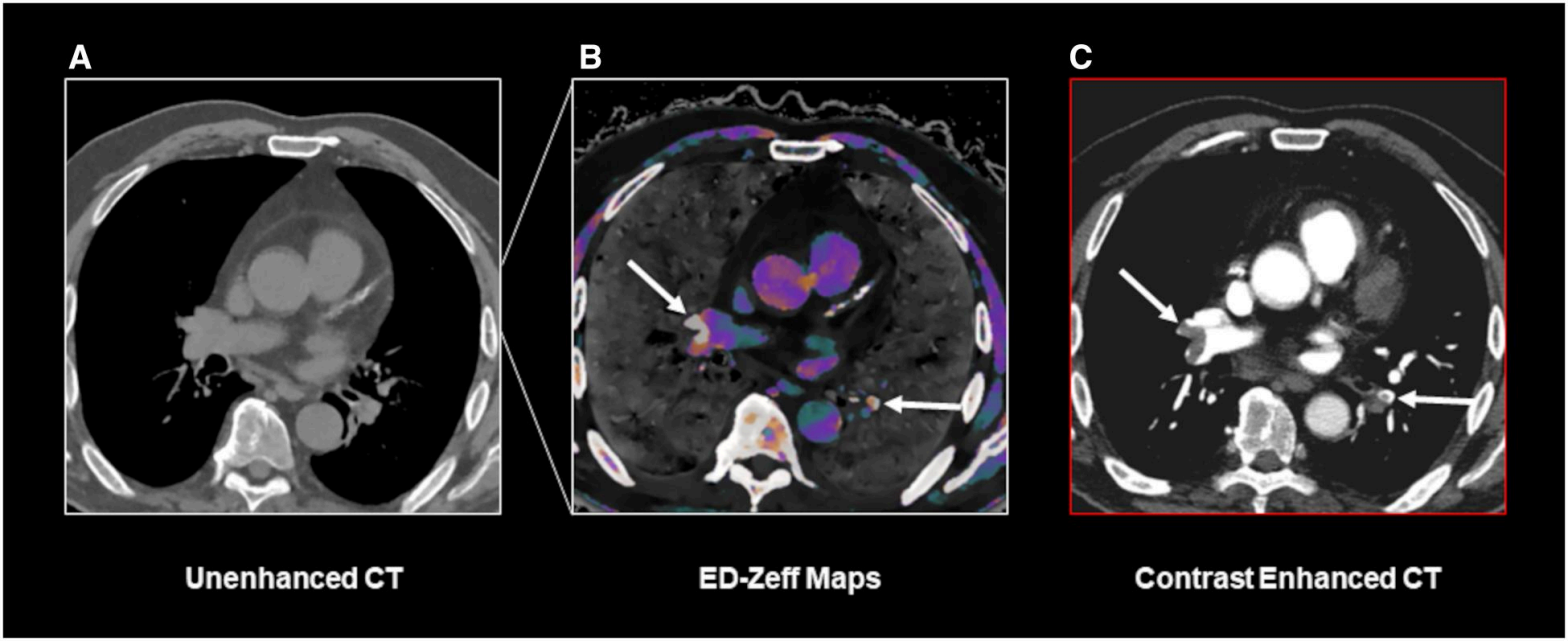
A true positive case of PE identified using the ED-Zeff (electron density-Z-effective) map. A 70-year-old male patient presenting to our department with chest pain and dyspnea. Unenhanced CT scan of the chest (A) shows no indirect signs of pulmonary embolism. ED-Zeff map (B), derived from the unenhanced CT scan, shows the presence of high signal spots at the bifurcation of right segmental pulmonary arteries and within a left segmental pulmonary artery (arrows). The subsequently performed CECT (contrast-enhanced CT) (C) confirms the presence of pulmonary emboli (arrows) at the same location as shown in B.

While our study was performed on a spectral CT scanner, to the best of our knowledge, reconstructions similar to ED-Zeff maps can also be obtained from DECT scanners with dual-source or kVp-switching configurations, as well as from photon-counting detector CT scanners.^28–30^ Future studies on larger cohorts are required to determine whether the diagnostic performance we report is generalizable to other scanner types. If so, in selected clinical scenarios, such as patients with severe renal impairment or proven allergies to contrast media, ED-Zeff maps may serve as a valuable tool. As DECT scanners are readily available in most urgent care settings, they can be used alongside pre-test probability and D-dimer testing, these maps may help rule out PE (Figures 3 and 4). In addition, studies correlating ED-Zeff maps with D-dimer levels and comparing them with ventilation/perfusion lung scintigraphy are warranted to assess their diagnostic utility in patients with contraindications to contrast media.

**Figure 5.**
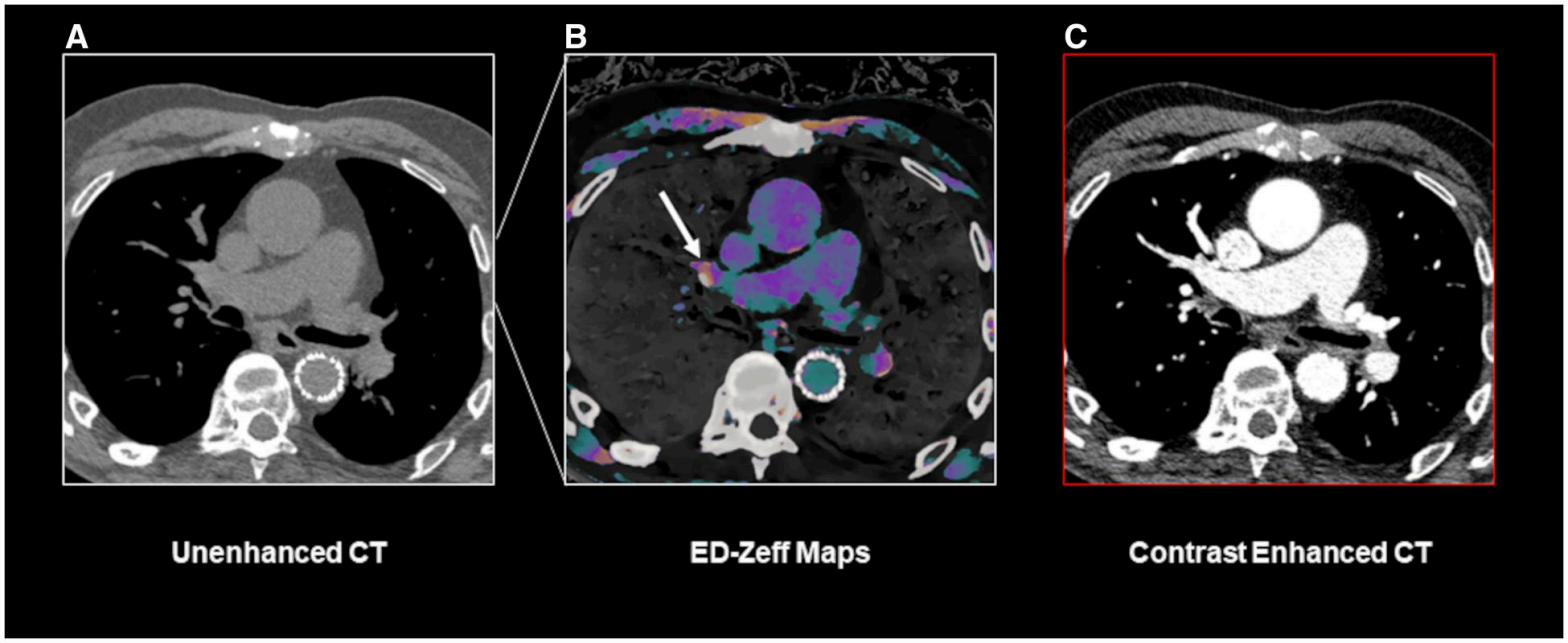
A false-positive case of PE identified using the ED-Zeff (electron density-Z-effective) map. An 82-year-old female patient presented to our department with chest pain. Unenhanced CT scan of the chest (A) shows no relevant vascular findings. ED-Zeff map (B), derived from the unenhanced CT scan, shows the presence of high signal spot within a right segmental artery (arrow). The subsequently performed CECT (contrast-enhanced CT) (C) shows no sign of pulmonary embolism.

**Figure 6.**
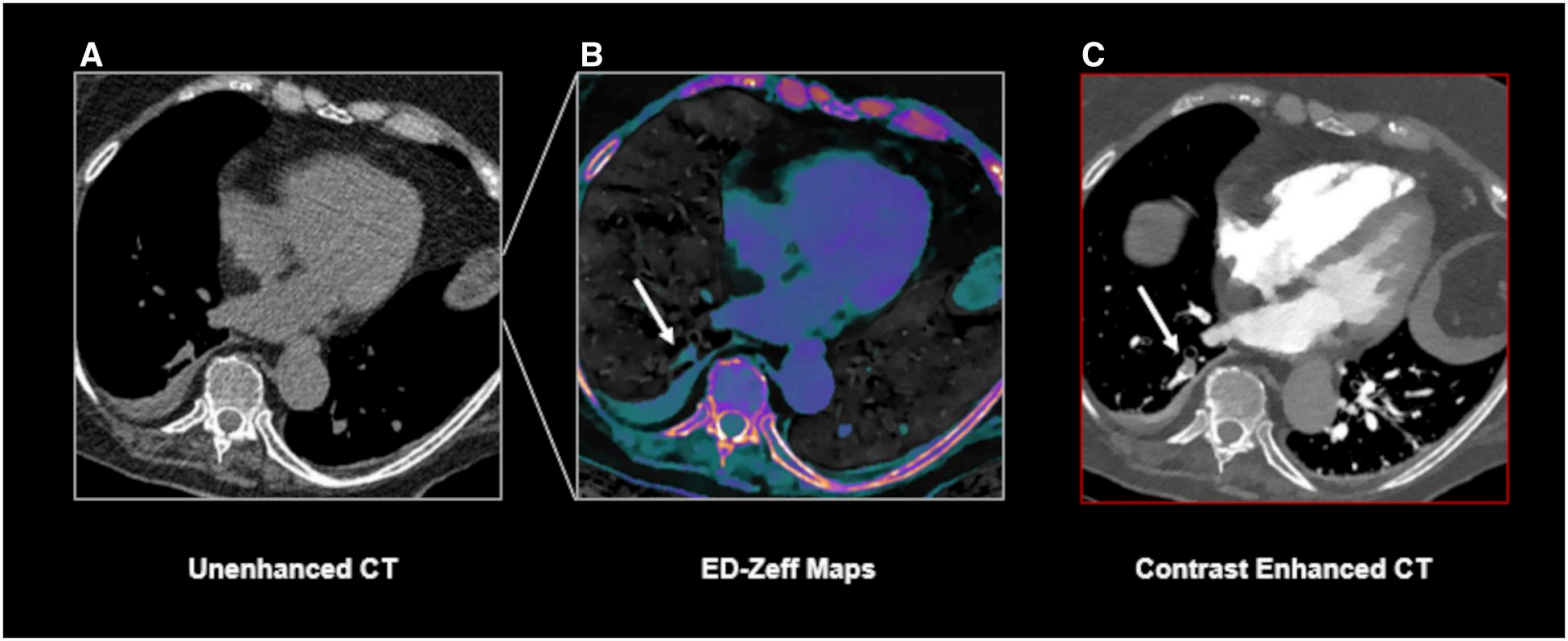
A false-negative case of PE identified using the ED-Zeff (electron density-Z-effective) map. A 69-year-old male patient presented to our department with dyspnea. Unenhanced CT scan of the chest (A) shows no relevant findings. ED-Zeff map (B), derived from the unenhanced CT scan, does not show any luminal difference of signal within a right segmental artery (arrow). The subsequently performed CECT (contrast-enhanced CT) revealed the presence of a sub-segmental pulmonary embolus.

This study has several limitations that need to be addressed. Firstly, the absence of a pulmonary artery-triggered CECT for all patients may reduce the strength of the reference standard. However, we optimized pulmonary artery visualization using 40 keV virtual monoenergetic reconstructions and included only patients with a pulmonary artery trunk attenuation higher than 250 HU. Secondly, our patient cohort is relatively small and from a single center, with a low prevalence of true positive PE cases. This may result in PPV and NPV that differ from those in a real-world scenario (Figures 5 and 6). Further studies involving larger patient cohorts may be needed to obtain more accurate results. Thirdly, we did not correlate with D-dimer values, which could potentially increase the diagnostic accuracy of this technique.^31^^,^^32^ Finally, we assessed the diagnostic performance based on images obtained from spectral dl-DECT, while alternative CT platforms or scanner configurations may yield different results.

In conclusion, our data demonstrate robust diagnostic accuracy of non-contrast ED-Zeff maps, indicating a high NPV in detecting PE. This suggests the potential for excluding PE by means of DECT, without the need of using contrast media. However, further investigations involving larger datasets and other scanner types are imperative to assess their clinical utility and generalizability in patients with low clinical suspicion of PE and contraindications to contrast media administration.

## Supplementary Material

umae025_Supplementary_Data

## Data Availability

Data generated or analyzed during the study are available from the corresponding author by request.

## References

[1] Henzler T, Barraza JM Jr, Nance JW Jr, et al CT imaging of acute pulmonary embolism. J Cardiovasc Comput Tomogr. 2011;5(1):3-11. 21051309 10.1016/j.jcct.2010.10.001

[2] Hong YJ, Shim J, Lee SM, Im DJ, Hur J. Dual-energy CT for pulmonary embolism: current and evolving clinical applications. Korean J Radiol. 2021;22(9):1555-1568. 34448383 10.3348/kjr.2020.1512 PMC8390816

[3] Yuan R, Shuman WP, Earls JP, et al Reduced iodine load at CT pulmonary angiography with dual-energy monochromatic imaging: comparison with standard CT pulmonary angiography—a prospective randomized trial. Radiology. 2012;262(1):290-297. 22084206 10.1148/radiol.11110648

[4] Dong J, Wang X, Jiang X, et al Low-contrast agent dose dual-energy CT monochromatic imaging in pulmonary angiography versus routine CT. J Comput Assist Tomogr. 2013;37(4):618-625. 23863541 10.1097/RCT.0b013e31828f5020

[5] Delesalle MA, Pontana F, Duhamel A, et al Spectral optimization of chest CT angiography with reduced iodine load: experience in 80 patients evaluated with dual-source, dual-energy CT. Radiology. 2013;267(1):256-266. 23319663 10.1148/radiol.12120195

[6] Stein PD, Fowler SE, Goodman LR, et al; PIOPED II Investigators. Multidetector computed tomography for acute pulmonary embolism. N Engl J Med. 2006;354(22):2317-2327. 16738268 10.1056/NEJMoa052367

[7] D'Angelo T, Cicero G, Mazziotti S, et al Dual energy computed tomography virtual monoenergetic imaging: technique and clinical applications. Br J Radiol. 2019;92(1098):20180546. 30919651 10.1259/bjr.20180546 PMC6592074

[8] Arico' FM, Trimarchi R, Portaluri A, et al Virtual monoenergetic dual-layer dual-energy CT images in colorectal cancer: CT diagnosis could be improved? Radiol Med. 2023;128(8):891-899. 37310558 10.1007/s11547-023-01663-0

[9] Rassouli N, Etesami M, Dhanantwari A, Rajiah P. Detector-based spectral CT with a novel dual-layer technology: principles and applications. Insights Imaging. 2017;8(6):589-598. 28986761 10.1007/s13244-017-0571-4 PMC5707218

[10] Rajiah P, Parakh A, Kay F, Baruah D, Kambadakone AR, Leng S. Update on multienergy CT: physics, principles, and applications. Radiographics. 2020;40(5):1284-1308. 32822281 10.1148/rg.2020200038

[11] Yang M, Virshup G, Clayton J, Zhu XR, Mohan R, Dong L. Theoretical variance analysis of single- and dual-energy computed tomography methods for calculating proton stopping power ratios of biological tissues. Phys Med Biol. 2010;55(5):1343-1362. 20145291 10.1088/0031-9155/55/5/006

[12] Mei K, Ehn S, Oechsner M, et al Dual-layer spectral computed tomography: measuring relative electron density. Eur Radiol Exp. 2018;2:20. 30175319 10.1186/s41747-018-0051-8 PMC6103960

[13] van Elmpt W, Landry G, Das M, Verhaegen F. Dual energy CT in radiotherapy: current applications and future outlook. Radiother Oncol. 2016;119(1):137-144. 26975241 10.1016/j.radonc.2016.02.026

[14] Nelles C, Lennartz S. Spinal hematoma visualized with dual-energy CT-derived electron density overlay maps. Radiology. 2023;307(4):e222680. 37039691 10.1148/radiol.222680

[15] Tatsugami F, Higaki T, Nakamura Y, Honda Y, Awai K. Dual-energy CT: minimal essentials for radiologists. Jpn J Radiol. 2022;40(6):547-559. 34981319 10.1007/s11604-021-01233-2 PMC9162973

[16] Schneider U, Pedroni E, Lomax A. The calibration of CT Hounsfield units for radiotherapy treatment planning. Phys Med Biol. 1996;41(1):111-124. 8685250 10.1088/0031-9155/41/1/009

[17] Mochizuki J, Nakaura T, Hashimoto K, Hata Y. Detection of ventricular thrombi via electron density imaging in non-contrast spectral computed tomography performed to exclude pneumonia: a case report. Eur Heart J Case Rep. 2022;6(4):ytac148. 35475194 10.1093/ehjcr/ytac148 PMC9024094

[18] Mochizuki J, Nakaura T, Matsumi H, Hata Y. Evaluation of coronavirus-2019-related arterial thrombosis in noncontrast spectral computed tomography with electron density imaging. Radiol Case Rep. 2023;18(1):49-52. 36317095 10.1016/j.radcr.2022.09.085 PMC9612951

[19] Bae K, Jeon KN. Diagnosis of pulmonary embolism in unenhanced dual energy CT using an electron density image. Diagnostics (Basel). 2021;11(10):1841. 34679538 10.3390/diagnostics11101841 PMC8534653

[20] Bae KT, Tao C, Gürel S, et al Effect of patient weight and scanning duration on contrast enhancement during pulmonary multidetector CT angiography. Radiology. 2007;242(2):582-589. 17255426 10.1148/radiol.2422052132

[21] Ramadan SU, Kosar P, Sonmez I, Karahan S, Kosar U. Optimisation of contrast medium volume and injection-related factors in CT pulmonary angiography: 64-slice CT study. Eur Radiol. 2010;20(9):2100-2107. 20437179 10.1007/s00330-010-1782-y

[22] D'Angelo T, Bucher AM, Lenga L, et al Optimisation of window settings for traditional and noise-optimised virtual monoenergetic imaging in dual-energy computed tomography pulmonary angiography. Eur Radiol. 2018;28(4):1393-1401. 29018926 10.1007/s00330-017-5059-6

[23] Bae K, Jeon KN, Cho SB, et al Improved opacification of a suboptimally enhanced pulmonary artery in chest CT: experience using a dual-layer detector spectral CT. AJR Am J Roentgenol. 2018;210(4):734-741. 29446668 10.2214/AJR.17.18537

[24] D'Angelo T, Lanzafame LRM, Micari A, et al Improved coronary artery visualization using virtual monoenergetic imaging from dual-layer spectral detector CT angiography. Diagnostics (Basel). 2023;13(16):2675. 37627934 10.3390/diagnostics13162675 PMC10453590

[25] New PF, Aronow S. Attenuation measurements of whole blood and blood fractions in computed tomography. Radiology. 1976;121(3 Pt. 1):635-640. 981659 10.1148/121.3.635

[26] Kirchhof K, Welzel T, Mecke C, Zoubaa S, Sartor K. Differentiation of white, mixed, and red thrombi: value of CT in estimation of the prognosis of thrombolysis phantom study. Radiology. 2003;228(1):126-130. 12728185 10.1148/radiol.2273020530

[27] Shrimpton PC. Electron density values of various human tissues: in vitro Compton scatter measurements and calculated ranges. Phys Med Biol. 1981;26(5):907-911. 7291311 10.1088/0031-9155/26/5/010

[28] Hu G, Niepel K, Risch F, et al Assessment of quantitative information for radiation therapy at a first-generation clinical photon-counting computed tomography scanner. Front Oncol. 2022;12:970299. 36185297 10.3389/fonc.2022.970299 PMC9515409

[29] Bharati A, Mandal SR, Gupta AK, et al Development of a method to determine electron density and effective atomic number of high atomic number solid materials using dual-energy computed tomography. J Med Phys. 2019;44(1):49-56. 30983771 10.4103/jmp.JMP_125_18 PMC6438052

[30] Kawahara D, Ozawa S, Yokomachi K, et al Evaluation of raw-data-based and calculated electron density for contrast media with a dual-energy CT technique. Rep Pract Oncol Radiother. 2019;24(5):499-506. 31467491 10.1016/j.rpor.2019.07.013 PMC6710634

[31] Gotta J, Koch V, Geyer T, et al Imaging-based risk stratification of patients with pulmonary embolism based on dual-energy CT-derived radiomics. Eur J Clin Invest. 2024;54(4):e14139. 38063028 10.1111/eci.14139

[32] Gotta J, Gruenewald LD, Geyer T, et al Indicators for hospitalization in acute pulmonary embolism: uncover the association between D-dimer levels, thrombus volume and radiomics. Acad Radiol. 2024;31(6):2610-2619. 38242733 10.1016/j.acra.2023.12.045

