# Supplementary material for: Pulmonary embolism detection without intravenous contrast using electron density and Z-effective maps from dual-energy CT
Source: Radiol Adv. 2024 Oct 24;1(3):umae025. doi: 10.1093/radadv/umae025 (PMC12429230; doi:10.1093/radadv/umae025)
Supplement: umae025_Supplementary_Data [file umae025_Supplementary_Data.zip › R1_Supplemental Figure.pdf]

SUPPLEMENTAL FIGURE

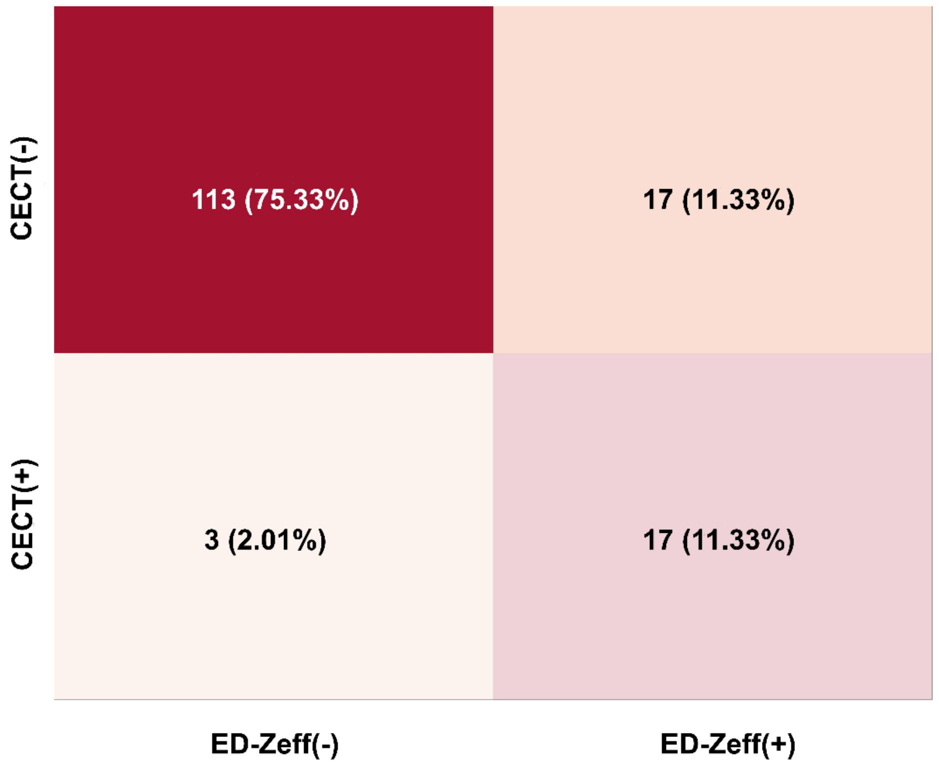

Figure S1*. Confusion matrix showing the results obtained by the most experienced reader to confirm or exclude the presence of pulmonary embolism on ED-Zeff maps. True Negatives= 113 (75.33%) in red box, True Positives= 17(11.33%) in pink box, False Positives= 17 (11.33%) in orange box, False Negatives= 3 (2.01%) in white box.*
